# Risk factors for sepsis mortality: A national Swedish cohort study (1998–2018)

**DOI:** 10.1016/j.clinsp.2026.101022

**Published:** 2026-06-10

**Authors:** Filip Jansåker, Xinjun Li, Per-Ola Forsberg, Kristina Sundquist, Henning Stenberg

**Affiliations:** a Center for Primary Health Care Research, Department of Clinical Sciences Malmö, Lund University, Malmö, Sweden; b University Clinic Primary Care, Skåne University Hospital, Region Skåne, Sweden; c Department of Clinical Microbiology, Center of Diagnostic Investigations, Rigshospitalet, Copenhagen University Hospital, Copenhagen, Denmark; d Department of Family and Community Medicine, McGovern Medical School, The University of Texas Health Science Center, Houston, TX, USA

**Keywords:** Epidemiology, Sepsis, Mortality, Sociodemographic factors, Sweden

## Abstract

**Objective:**

Large population-based studies that could identify risk factors associated with sepsis mortality are scarce, but could be helpful in clinical risk assessments. With this study, the authors aimed to examine possible risk factors for sepsis mortality.

**Methods:**

The authors conducted an open nationwide cohort study, including 157,143 individuals aged ≥ 18-years diagnosed with (first event) sepsis (1998–2018). The outcome was 30-day all-cause mortality following sepsis. The predictors were sociodemographic factors, the Charlson Comorbidity Index, severe mental disorders, pyelonephritis and influenza/pneumonia diagnosis within 30-days before sepsis. Logistic regression models were used to calculate Odds Ratios (OR) and 95% Confidence Intervals (95% CI).

**Results:**

The overall 30-day mortality proportion was 17.9%, and 30.5% among those aged ≥ 85-years. In the full model, several sociodemographic factors were associated with mortality. For example, individuals aged ≥ 85-years had higher mortality (OR = 13.02, 95% CI 11.50–14.74) compared to those aged 18–44 years. Mortality was also higher among individuals with low educational level (1.09, 1.05–1.13), with low family income (1.17, 1.14–1.21), living outside large cities (1.09, 1.06–1.12), and not being married/cohabiting (1.17, 1.14–1.21), whereas individuals not born in Sweden (0.93, 0.89–0.98) had slightly lower mortality, compared to their respective reference category. Pyelonephritis was associated with lower mortality (0.25, 0.22–0.28) compared with no preceding diagnosis, whereas influenza/pneumonia had no significant association with mortality (1.06, 0.99–1.13).

**Conclusion:**

These findings can be used to increase the awareness of potential risk factors for sepsis mortality in all settings where patients with sepsis are treated and subsequently followed.

## Highlights

• This nationwide open cohort study examined risk factors for sepsis mortality.
• Age and several other sociodemographic factors were associated with mortality.
• A preceding pyelonephritis diagnosis was associated with lower mortality.

## Introduction

Sepsis is a severe disease caused by a dysregulated host response to an infection (mainly bacterial), leading to systemic inflammation and, in many cases, multiple organ dysfunction and death.^1^ The 30-day mortality of sepsis is estimated to be over 20%, and almost one in three deaths occurring in hospitals is due to sepsis.^2^, ^3^, ^4^ Identifying risk factors for sepsis mortality is important to help clinicians identify patients who are at increased risk.

Previous research has found that sepsis mortality increases considerably with age and is higher among those with preexisting comorbidities.^5^, ^6^, ^7^ Although men have a higher risk of developing sepsis,^8^ the results from different studies are inconsistent regarding sex differences in sepsis mortality.^9^ Other sociodemographic factors have also been associated with sepsis incidence^6^, ^8^, ^10^ and studies from the United States (US) have shown that the mortality is almost twice as high for non-white compared to white patients with sepsis.^6^^,^^11^ Moreover, while severe mental disorder has been found to be a considerable risk factor for sepsis incidence,^8^^,^^12^ a recent study from the US indicated that the mortality in patients hospitalized for sepsis seems to be lower in those with preexisting mental disorders.^13^ Additionally, respiratory and urinary tract infections are common causes of sepsis^14^^,^^15^ and sepsis mortality seems to vary depending on microbiological findings in blood cultures; i.e., mortality tends to be higher with Gram-positive (common in respiratory tract infections) compared to Gram-negative sepsis (common in urinary tract infections).^16^ Influenza is also indirectly associated with severe sepsis by secondary bacterial infections, which are often Gram-positive.^17^ The mortality after sepsis might therefore also differ depending on which clinical infection (e.g., respiratory or urinary tract infection) preceded the sepsis event.

Large-scale population-based studies, including individual-level sociodemographic and clinical data from hospitals and primary healthcare settings, examining risk factors for sepsis mortality are warranted.^6^^,^^11^ While studies from the US have linked sociodemographic factors to sepsis mortality, sociodemographic determinants of sepsis mortality remain largely unexplored in large-scale cohorts from diverse populations with access to universal healthcare systems. Sweden is an ideal setting to conduct such studies, due to its universal healthcare system,^18^ comprehensive national registers^19^, ^20^ and primary healthcare data,^21^ and unique personal identification numbers,^22^ which enable linkage of individual-level data from different sources. With this nationwide study, including all adults in the Swedish population diagnosed with sepsis during a 21-year time period, the authors aimed to examine possible risk factors for 30-day sepsis mortality.

## Material and methods

### Study design and setting

A nationwide open cohort study including individuals aged ≥ 18-years diagnosed with sepsis in Sweden between January 1, 1998, and October 1, 2018, was conducted. National registers and primary healthcare data were used in this study. At birth or upon immigration, all individuals residing in Sweden are assigned a unique 10-digit personal identification number. This number is utilized at all healthcare contacts and for the collection of data to national registers by the public authorities, thus ensuring practically full coverage of Sweden’s population and minimal loss of follow-up.^22^

### Study population

The authors identified a total of 157,143 adult individuals with a sepsis or septic shock diagnosis (first event) registered during the study period. The 10th revision of the International Classification of Diseases (ICD-10) coded diagnoses of sepsis or septic shock registered between January 1, 1998, and October 1, 2018, i.e., ≈90-days before the end of the study period (December 31, 2018), were used to identify cases. The ICD-10 code A41 (“Other sepsis”; 80.8%) comprised the majority of all cases, followed by A40 (“Sepsis due to streptococcus”; 13.0%). The frequency of the other sepsis diagnoses (6.2% in total) ranged from 0.0% to 4.2% (Supplementary Table S1). Septic shock (ICD-10 code R57.2), comprising 1.7% of all cases (n = 2627), was excluded from the main analyses and analyzed separately. In total, 154,516 individuals with a sepsis diagnosis were included in the main analysis (Fig. S1). The study did not include pregnancy-, pediatric/neonatal-, and procedure- (iatrogenic) related sepsis diagnoses.

### Outcome

The outcome was all-cause mortality after the first sepsis diagnosis (hereafter called sepsis mortality). In the main analyses, the outcome was mortality within 30-days (30-day mortality) after sepsis diagnosis. Mortality within 90-days (90-day mortality) after sepsis was used as the outcome in a sensitivity analysis.

### Predictors

Sociodemographic (individual-level) factors were collected at baseline (i.e., time of sepsis diagnosis) and included sex, age, educational level, family income, region of residence, marital status, and country of origin. *Sex* was categorized as male or female. *Age* was categorized into the following groups: 18–44, 45–64, 65–84, or ≥85-years of age. *Educational level* was categorized into two groups based on the duration of school years attended: compulsory education with or without completed high school education (≤ 12-years) or at least one year of university or college education (>12-years). *Family income* was based on a weighted average income in each family and categorized into low (below the median family income level of the study population) and high (above the median). *Region of residence* was categorized into two groups: residing in the largest cities (Malmö, Gothenburg, or Stockholm) or outside of the largest cities. *Marital status* was categorized as married/cohabiting or unmarried/divorced/widowed. *Country of origin* was categorized as born in Sweden or born outside of Sweden (foreign-born).

*Charlson Comorbidity Index* (CCI) was used as a measure for comorbidities, categorized as low (0p), moderate (1‒2p), or high (≥ 3) CCI-scores.^23^ The CCI was constructed and adapted for register-based research in Sweden^24^ and based on recorded CCI diagnosis codes within a five-year period prior to the baseline (Supplementary Table S2). *Severe mental disorders,* which are not included in the CCI, were measured as an ICD-10 coded diagnosis of any psychotic disorder (F20, F22, and F25) or bipolar/single manic episode (F30 and F31). The authors also included diagnoses of influenza and pneumonia or pyelonephritis as proxies for the potential clinical sources of sepsis in a sub-analysis. *Influenza/pneumonia* was measured as an ICD-10 code of J09–J18, and *pyelonephritis* as an ICD-10 code of N10 or N12 at any time within 30-days prior to the sepsis event.

### Data sources

The register used to identify the study population was the National Patient Register (NPR),^19^ the NPR (i.e., both inpatient and outpatient data) and population-based primary healthcare data from 20 out of 21 administrative healthcare regions in Sweden^21^ were used to collect data on preceding infections, severe mental disorders, and comorbidities for the CCI. The Total Population^20^ was used to collect data on the sociodemographic variables. The Swedish Cause of Death Register^25^ was used to identify the outcome. All linkages between the registry data were performed using a pseudonymized version of the personal identification number.^22^ Individuals with missing observations for any sociodemographic variable (n = 3209) were excluded from the main analyses, i.e., 2.0% of the study population. Missing values were found for educational level, family income, and region of residence, and were most prevalent in those born before 1960 (n = 2413).

### Statistical analysis

Descriptive statistics and 30-day mortality proportions were calculated for each predictor variable. Logistic regression models were used to estimate the association between the predictors and sepsis mortality. The results are presented as Odds Ratios (OR) and 95% Confidence Intervals (95%CI). Three models were used: Model 1, a univariable model; Model 2, an age-adjusted model; and Model 3, a full model including age, sex, all sociodemographic variables, comorbidities, and severe mental disorders. The authors also conducted several additional analyses to test the robustness of the main findings and to examine whether the associations differed across subgroups. First, given that age is a considerable risk factor for sepsis mortality and that age is associated with the other predictor variables, the authors conducted a fully adjusted analysis in adults aged ≥ 45-years with sepsis to compare the associations with those in the main analysis of all adults. Second, as the country of origin is associated with the other predictors, the authors conducted a fully adjusted analysis stratified by country of origin to assess whether the associations varied between patients born in Sweden and foreign-born patients. Third, variations occurred over the study period in diagnostic procedures and sepsis definitions. Therefore, the authors conducted a fully adjusted sensitivity analysis stratified by the time period of the first sepsis diagnosis to assess whether the associations were consistent across the study period. The time periods were categorized into 1998–2005, 2006–2012, and 2013–2018. An additional fully adjusted analysis, including the potential clinical source of infection, was also conducted to assess whether the clinical source of infection was associated with sepsis mortality and might have affected the main findings. The authors also conducted a fully adjusted sensitivity analysis for septic shock, as this condition is distinct from sepsis. Furthermore, a fully adjusted sensitivity analysis on 90-day mortality was conducted to examine whether the associations remained during longer follow-up. Finally, a sensitivity analysis, including missing values as a separate group, was conducted to assess whether the main findings were affected by missing data. A two-tailed p-value of < 0.05 was used to determine statistical significance. Statistical formulas are included in the Supplementary Material. SAS software version 9.4 (SAS Institute Inc.; Cary, NC, USA) was used for all statistical analyses.

### Ethical statement

The present study was a non-intervention nationwide register study based on pseudonymized secondary data collected by and obtained from Swedish authorities and was approved for use by the Ethical Review Board in Lund (Dnr. 2012/795 and later approved amendments), Sweden. Use of secondary, nationwide data obtained from the Swedish authorities does not require informed consent.

## Results

Table 1 shows the descriptive characteristics of the study population, consisting of 154,516 patients diagnosed with sepsis (first event) during the study period (1998–2018). The overall 30-day sepsis mortality proportion was 17.9% (95% CI 17.7–18.2) and increased from 3.1% (95% CI 2.8–3.5) in those aged 18–44 years to 30.5% (95% CI 29.9–31.0) in those aged ≥ 85-years. Supplementary Table S3 shows the number of patients and 30-day mortality proportions in different time periods of sepsis diagnosis (1998–2005, 2006–2012, and 2013–2018) and for the subgroup diagnosed with septic shock (n = 2627), in which the 30-day mortality was 53.8%.

**Table 1:** Study population of patients diagnosed with sepsis (first event) and 30-day all-cause mortality (1998–2018).

	Study population		30-day mortality		30-day mortality per 100 persons		
	No.	%	No.	%	%	95% CI	
**Total population**	154,516		27,722		17.9	17.7	18.2
**Sex**							
Male	86,517	56.0	15,272	55.1	17.7	17.4	17.9
Female	67,999	44.0	12,450	44.9	18.3	18.0	18.6
**Age at baseline (years)**							
18–44	8576	5.6	269	1.0	3.1	2.8	3.5
45–64	30,838	20.0	2701	9.7	8.8	8.4	9.1
65–84	81,205	52.6	14,427	52.0	17.8	17.5	18.1
≥ 85	33,897	21.9	10,325	37.2	30.5	29.9	31.0
**Educational level (years)**							
≤ 12	126,186	81.7	23,230	83.8	18.4	18.2	18.6
> 12	28,330	18.3	4492	16.2	15.9	15.4	16.3
**Family income**							
Low	115,992	75.1	21,897	79.0	18.9	18.6	19.1
High	38,524	24.9	5825	21.0	15.1	14.7	15.5
**Region of residence**							
Large cities	80,988	52.4	14,058	50.7	17.4	17.1	17.6
Others	73,528	47.6	13,664	49.3	18.6	18.3	18.9
**Marital status**							
Married/cohabiting	86,759	56.1	15,113	54.5	17.4	17.1	17.7
Unmarried/Divorced/ Widowed	67,757	43.9	12,609	45.5	18.6	18.3	18.9
**Country of origin**							
Sweden	139,028	90.0	25,351	91.4	18.2	18.0	18.5
Foreign	15,488	10.0	2371	8.6	15.3	14.7	15.9
**Charlson Comorbidity Index**							
Low (0 p)	85,811	55.5	13,090	47.2	15.3	15.0	15.5
Moderate (1–2 p)	67,778	43.9	14,430	52.1	21.3	20.9	21.6
High (≥ 3 p)	927	0.6	202	0.7	21.8	18.8	24.8
**Severe mental disorders**							
No diagnosis	151,742	98.2	27,353	98.7	18.0	17.8	18.2
Diagnosis	2774	1.8	369	1.3	13.3	11.9	14.7

Table 2 shows the results of the main analysis. In the univariable model 1, all predictors were associated with 30-day sepsis mortality. The association between age and 30-day mortality was particularly strong, with an OR of 13.52 (95% CI 11.95–15.30) in individuals aged ≥ 85-years compared to those aged 18–44-years. In both the age-adjusted and full model (models 2–3), sex and severe mental disorders were no longer significantly associated with mortality, while the other associations remained significant but were somewhat altered for some covariates. For example, the association between educational level and mortality was attenuated, whereas the association between marital status and mortality increased. Table 3 (excluding the youngest age group, aged 18–44 years) shows results that were similar to the main analysis. The findings also persisted in the analysis stratified by country of origin, except for family income, which was not associated with mortality in foreign-born patients (Supplementary Table S4). The sensitivity analysis, including those with missing values (n = 3209) for educational level, family income, and region of residence (data not shown), also yielded results similar to the main analysis.

**Table 2:** Association of individual sociodemographic factors and comorbidities with 30-day all-cause sepsis mortality (1998–2018).

	Model 1				Model 2				Model 3			
Covariates	OR	95% CI		p-value	OR	95% CI		p-value	OR	95% CI		p-value
**Age** (ref. age 18–44 years)												
45–64	2.96	2.61	3.37	<0.0001	2.96	2.61	3.37	<0.0001	3.02	2.66	3.43	<0.0001
65–84	6.67	5.90	7.54	<0.0001	6.67	5.90	7.54	<0.0001	6.61	5.84	7.47	<0.0001
≥ 85	13.52	11.95	15.30	<0.0001	13.52	11.95	15.30	<0.0001	13.02	11.50	14.74	<0.0001
**Male sex** (ref. female)	0.96	0.93	0.98	0.0001	0.99	0.96	1.01	0.3191	1.02	0.99	1.04	0.2751
**Educational level** (ref. > 12 years)	1.20	1.16	1.24	<0.0001	1.16	1.12	1.20	<0.0001	1.09	1.05	1.13	<0.0001
**Family income** (ref. high)	1.31	1.27	1.35	<0.0001	1.20	1.16	1.24	<0.0001	1.17	1.14	1.21	<0.0001
**Region of residence** (ref. large cities)	1.09	1.06	1.12	<0.0001	1.09	1.07	1.12	<0.0001	1.09	1.06	1.12	<0.0001
**Marital status** (ref. married/cohabiting)	1.08	1.06	1.11	<0.0001	1.16	1.13	1.19	<0.0001	1.17	1.14	1.21	<0.0001
**Country of origin** (ref. Sweden)	0.81	0.78	0.85	<0.0001	0.92	0.88	0.97	0.001	0.93	0.89	0.98	0.0038
**Charlson Comorbidity Index** (ref. low, 0 p)												
Moderate (1–2 p)	1.50	1.46	1.54	<0.0001	1.33	1.30	1.37	<0.0001	1.33	1.30	1.37	<0.0001
High (≥ 3 p)	1.55	1.32	1.81	<0.0001	1.86	1.58	2.18	<0.0001	1.94	1.65	2.28	<0.0001
**Severe mental disorders** (ref. no diagnosis)	0.70	0.63	0.78	<0.0001	0.97	0.86	1.08	0.5641	0.94	0.84	1.05	0.2784

**Table 3:** Association of individual sociodemographic factors and comorbidities with 30-day all-cause sepsis mortality, after excluding individuals aged 18–44 years.

Covariates	OR	95% CI		p-value
**Age** (ref. age 45–64 years)				
65–84	2.19	2.10	2.29	<0.0001
≥ 85	4.32	4.12	4.52	<0.0001
**Male sex** (ref. female)	1.01	0.98	1.04	0.2751
**Educational level** (ref. >12-years)	1.09	1.05	1.13	<0.0001
**Family income** (ref. high)	1.17	1.13	1.21	<0.0001
**Region of residence** (ref. large cities)	1.09	1.06	1.12	<0.0001
**Marital status** (ref. married/cohabiting)	1.17	1.14	1.20	<0.0001
**Country of origin** (ref. Sweden)	0.94	0.89	0.98	0.0038
**Charlson Comorbidity Index** (ref. low, 0 p)				
Moderate (1–2 p)	1.32	1.29	1.36	<0.0001
High (≥3 p)	1.87	1.59	2.20	<0.0001
**Severe mental disorders** (ref. no diagnosis)	0.95	0.85	1.07	0.2784

OR, Odds ratio; CI, Confidence interval. Full model, adjusted for all covariates. Analysis excluding those diagnosed with septic shock (n = 2627).

Table 4 shows results from full models stratified by time periods of the first event, sepsis diagnosis. Most associations were consistent and similar to the main findings across the different time periods, albeit with a few notable exceptions. First, the association between age and 30-day mortality appeared weaker in 2013–2018, with an OR of 6.21 (95% CI 4.06–9.49) in individuals aged ≥ 85-years compared to those aged 18–44 years. Second, the association between sex and mortality was significant in 1998–2005, with a slightly elevated OR of 1.06 (95% CI 1.01–1.11) in men compared to women. Third, the association between education level and mortality was not significant in 1998–2005. Lastly, no significant association was observed for country of origin in 2013–2018, while a significant association was observed between severe mental disorder and 30-day sepsis mortality (OR = 0.78, 95% CI 0.62–0.98) in 1998–2005.

**Table 4:** Association of individual sociodemographic factors and comorbidities with 30-day all-cause sepsis mortality, stratified by time periods (calendar years) of first sepsis diagnosis during the study period.

	Period: 1998–2005				Period: 2006–2012					Period: 2013–2018			
Covariates	OR*	95% CI		p-value	OR*	95% CI		p-value		OR*	95% CI		p-value
**Age** (ref. age 18–44 years)													
45–64	3.07	2.57	3.65	<0.0001	3.07	2.48	3.80		<0.0001	1.74	1.13	2.69	0.0117
65–84	6.28	5.32	7.42	<0.0001	7.13	5.80	8.78		<0.0001	3.09	2.02	4.71	<0.0001
≥ 85	10.79	9.11	12.77	<0.0001	14.51	11.79	17.86		<0.0001	6.21	4.06	9.49	<0.0001
**Male sex** (ref. female)	1.06	1.01	1.11	0.0119	1.02	0.97	1.06		0.4824	0.98	0.92	1.04	0.5258
**Educational level** (ref. >12-years)	0.99	0.93	1.05	0.6416	1.09	1.03	1.16		0.0029	1.20	1.10	1.30	<0.0001
**Family income** (ref. high)	1.34	1.26	1.42	<0.0001	1.21	1.15	1.27		<0.0001	1.13	1.05	1.21	0.0011
**Region of residence** (ref. large cities)	1.09	1.04	1.14	0.0002	1.09	1.05	1.14		<0.0001	1.13	1.06	1.21	0.0001
**Marital status** (ref. married/cohabiting)	1.30	1.24	1.36	<0.0001	1.19	1.15	1.24		<0.0001	1.10	1.03	1.17	0.0072
**Country of Origin** (ref. Sweden)	0.88	0.81	0.95	0.0022	0.91	0.85	0.98		0.0106	1.00	0.90	1.11	0.9650
**Charlson Comorbidity Index** (ref. low, 0 p)													
Moderate (1–2 p)	1.41	1.35	1.47	<0.0001	1.45	1.39	1.51		<0.0001	1.12	1.05	1.19	0.0010
High (≥ 3 p)	1.64	1.12	2.39	0.0104	2.25	1.80	2.80		<0.0001	1.43	1.06	1.94	0.0209
**Severe mental disorders** (ref. no diagnosis)	0.78	0.62	0.98	0.0319	0.99	0.86	1.16		0.9393	0.97	0.74	1.26	0.7984

Table 5 shows the results from a full model, including the potential source of sepsis in the analysis. The findings show that having been diagnosed with pyelonephritis within 30-days before the sepsis event was associated with a significantly lower 30-day sepsis mortality (OR = 0.25, 95% CI 0.22–0.28) compared with no preceding diagnosis. The association between a preceding influenza/pneumonia diagnosis and sepsis mortality was non-significant, albeit indicative of slightly higher mortality (OR = 1.06, 95% CI 0.99–1.13), compared with no preceding diagnosis. The results for the sociodemographic factors, CCI, and severe mental disorders remained similar to the main findings, except for the country of origin, which was no longer significant.

**Table 5:** Association of individual sociodemographic factors and comorbidities with 30-day all-cause sepsis mortality, including preceding infections (diagnosed within 30-days before sepsis).

Covariates	OR	95% CI		p-value
**Age** (ref. age 18–44 years)				
45–64	3.01	2.65	3.42	<0.0001
65–84	6.63	5.86	7.51	<0.0001
≥ 85	13.14	11.61	14.88	<0.0001
**Male sex** (ref. female sex)	1.01	0.98	1.04	0.5792
**Educational level** (ref. >12-years)	1.09	1.05	1.13	<0.0001
**Family income** (ref. high)	1.17	1.13	1.21	<0.0001
**Region of residence** (ref. large cities)	1.10	1.07	1.13	<0.0001
**Marital status** (ref. married/cohabiting)	1.17	1.14	1.20	<0.0001
**Country of Origin** (ref. Sweden)	0.95	0.86	1.04	0.2598
**Charlson Comorbidity Index** (ref. no point)				
Moderate (1–2 p)	1.33	1.29	1.36	<0.0001
High (≥ 3 p)	1.92	1.64	2.26	<0.0001
**Severe mental disorders** (ref. no diagnosis)	0.94	0.84	1.05	0.2766
**Diagnosis of pyelonephritis within 30 days before sepsis** (ref. no diagnosis)	0.25	0.22	0.28	<0.0001
**Diagnosis of influenza/pneumonia within 30 days before sepsis** (ref. no diagnosis)	1.06	0.99	1.13	0.0897

OR, Odds Ratio; CI, Confidence Interval. Full model, adjusted for all covariates. Analysis excluding those diagnosed with septic shock (n = 2627).

Supplementary Table S5 shows the results from a sensitivity analysis in which risk factors associated with 30-day mortality were analyzed for those with septic shock. The associations appeared similar to the main findings but were only significant for age, marital status, and CCI (moderate vs. low).

Supplementary Table S6 shows the association between the predictors and 90-day sepsis mortality (excluding septic shock). In this analysis, the associations were more or less similar to the main analysis (Table 2), with the exception that men had a small but significant increase in 90-day mortality compared to women (OR = 1.03, 95% CI 1.01–1.06), and that those with a diagnosis of severe mental disorders had significantly lower sepsis mortality compared to those without severe mental disorders (OR=0.85, 95% CI 0.77–0.94).

## Discussion

In this nationwide cohort study, the authors examined risk factors for 30-day all-cause mortality following sepsis diagnosis in 154,516 adults. The overall mortality proportion was 17.9% in the total population and was particularly high (30.5%) among those aged ≥85-years. Several sociodemographic factors and CCI were associated with sepsis mortality, and old age was found to be a particularly strong risk factor. Patients with a preceding pyelonephritis had lower mortality, compared to those with no prior infection.

The findings that age and CCI were associated with sepsis mortality are consistent with previous studies^5^, ^6^, ^7^ and are likely explained by age- and disease-related impairments in immunological and cardiovascular functions. These findings suggest that advanced age and preexisting comorbidities are important risk factors for sepsis mortality. However, the associations appeared to be less pronounced in 2013–2018, which might reflect improvements in sepsis recognition and management in older patients and those with preexisting diseases, as well as improved care and treatment of chronic diseases. Moreover, similar to a previous study from Texas (US),^13^ the authors found that severe mental disorders were associated with lower sepsis mortality. However, in the time-stratified analysis, the association was significant only in 1998–2005 and, for the entire period, only in the univariable model. This may reflect that patients with mental disorders have been found to be younger and have fewer somatic comorbidities than other patients when diagnosed with sepsis.^13^ The weak and inconsistent associations between sex and sepsis mortality may indicate that sepsis care in Sweden (a high-income country with universal healthcare coverage) does not differ substantially by sex. Previous studies have, however, also reported inconsistent findings for the potential sex differences in sepsis mortality,^9^ suggesting that these particular findings may not be applicable in all other settings.

The association between marital status and sepsis mortality has previously not been reported and indicates that individuals who live alone have a higher risk of sepsis mortality. One possible explanation could be that individuals living alone may seek emergency care later than those who cohabit, thus potentially increasing their risk of delayed sepsis treatment. Differences in healthcare seeking patterns may also explain the observations that individuals of low socioeconomic status and those born in Sweden were found to have higher mortality after sepsis compared to individuals with high socioeconomic status and those born outside of Sweden. Another possible explanation for the increased mortality in individuals born in Sweden compared to those born in other countries could be the ‘healthy-migrant effect’,^26^ i.e., that individuals who migrate to other countries are, in general, healthier than those who stay in their country of birth (since higher morbidity can affect the ability to migrate). Moreover, a Danish study found that migrants had a higher risk of Gram-negative sepsis, but a lower risk of Gram-positive sepsis compared to non-migrants.^15^ Given that the mortality is higher in Gram-positive sepsis compared to Gram-negative sepsis,^16^ this may also be an explanation for the association between country of origin and mortality following sepsis in the present study, which disappeared after including preceding infections (potential infection source) in the analysis. However, the association between country of origin and sepsis mortality was not significant in 2013–2018, which may suggest that while this could have been a risk factor for sepsis mortality in Sweden in earlier years, it no longer appears to be so. Additionally, in contrast to Swedish-born individuals, no association between family income and sepsis mortality was observed among foreign-born individuals, suggesting that income-related differences in health may be less pronounced in this group. Recent research has also shown that income-related differences in all-cause mortality in Sweden is lower in the first migrant generation than in descendants of migrants.^27^

The finding that a clinical infection diagnosis preceding the sepsis diagnosis was associated with sepsis mortality in this population-based cohort is consistent with earlier reports of higher mortality in Gram-positive than Gram-negative sepsis,^16^ probably reflecting differences in sepsis sources. That is, a preceding diagnosis of pyelonephritis (mainly caused by Gram-negative bacteria, particularly *E. coli*) was associated with lower sepsis mortality, whereas influenza or pneumonia (often caused by Gram-positive bacteria, particularly *Streptococcus pneumoniae*) was indicative (non-significant) of slightly higher mortality. Additionally, severe sepsis after influenza could be caused directly by the virus-induced organ damage, secondary bacterial infections (Gram-positive or Gram-negative), or a combination of both.^17^ It is also possible that bacteriological cultures with antimicrobial susceptibility patterns may be available more often in patients with a preceding pyelonephritis (e.g. urine cultures) than in those with a preceding lower respiratory infection (e.g. sputum samples) at the time of sepsis diagnosis. However, as this study lacks microbiological data, the underlying mechanisms cannot be confirmed and need to be examined in detailed clinical studies.

There are some limitations of this study that need to be considered. First, as with most other large-scale register studies, it was limited to diagnosis data and did not have access to microbiological (e.g., blood cultures) or clinical data (e.g., symptoms, including the severity of the sepsis event) to validate the sepsis diagnoses. Therefore, it is possible that some cases were misdiagnosed as sepsis instead of another acute severe medical condition (e.g., severe organ dysfunction without confirmed infection). In addition, less severe confirmed infections, without sepsis criteria fulfilled, could also have been misdiagnosed as sepsis. Missed diagnosis of sepsis is another important potential limitation in research based on secondary data, and a recent meta-analysis has indicated that under-coding of sepsis exists.^28^ Although it is unclear whether the degree of misclassification varies across population groups and settings, it could be more pronounced in elderly and other at-risk populations, who may be more prone to having a missed sepsis diagnosis due to diagnostic difficulties and nonspecific symptoms.^29^ Second, the definition of sepsis varied across the study period,^1^ which could have affected the results depending on which time period the sepsis diagnosis was registered. Third, although the analyses adjusted for potential confounders, residual cofounding may persist, particularly for country of origin, where the associations could be explained by differences in health status,^26^ and type of infection (e.g., Gram-positive or Gram-negative sepsis).^15^ In an attempt to address these limitations, the authors performed several sensitivity analyses, and the results remained almost unchanged for most variables: one for septic shock, a distinct and more severe form of sepsis; one that excluded the youngest age category (individuals aged 18–44 years); one that stratified by time period (1998–2005, 2006–2012, and 2013–2018) of sepsis diagnosis; one that stratified by country of origin; and one on 90-day sepsis mortality. In addition, the associations between the sociodemographic factors and sepsis mortality remained when adjusting for preceding infections (except for the variable country of origin). Although including those with missing values (educational level, region of residency, and country of origin) did not change the main findings, missing values could potentially have biased the associations. Additionally, effects of unmeasured confounding may also exist. For example, the authors did not have access to tobacco smoking and social support networks could have confounded some of the findings, especially for marital status.^30^ Nevertheless, a large part of the effect that smoking would have on an individual’s ability to survive sepsis would likely be mediated through associated comorbidities, many of which were accounted for in this study by the use of CCI. Finally, certain potential confounders and mediators could not be assessed in the available dataset. For instance, socioeconomic status could influence care-seeking behavior, which in turn could affect the timing of presentation and mortality outcomes. Further studies are needed to examine these potential mechanisms in greater detail.

This study also has several strengths that balance the limitations. A major strength was the use of large population-based data from national registers of high completeness, spanning over a long time period (21-years), with little loss to follow-up. Furthermore, access to data from hospital and outpatient specialist care, as well as primary healthcare settings, enabled a more complete collection of comorbidity data, allowing us to construct a more comprehensive CCI than is generally possible. In addition, this also enabled us to include infections diagnosed before the sepsis events from all levels of healthcare, including primary healthcare, where most infections are diagnosed and treated. Moreover, the unique personal identification number enabled us to link sociodemographic variables with clinical diagnoses at an individual level to account for a larger number of potential confounders. Lastly, the high overall 30-day mortality proportion and other findings of ours were consistent with previous studies.^4^, ^5^, ^6^, ^7^^,^^13^ Additionally, although temporal changes in sepsis diagnosis criteria and care have occurred, most associations were consistent across the different time periods of the study period. Altogether, this shows that the national registers applied in this study can be used to identify sepsis diagnoses and assess potential risk factors for subsequent mortality, and that most of these findings are robust and reliable. However, studies including microbiological and clinical data (e.g., symptoms and severity) are needed to validate the present findings.

The present findings may have several clinical and public health implications. First, several sociodemographic factors were associated with sepsis mortality, with most associations being consistent over time and in the different analyses. Increased knowledge of these potential risk factors for sepsis mortality may be useful in clinical practice by guiding treatment and care for patients with sepsis during hospitalization. Moreover, the association between preceding pyelonephritis and sepsis mortality suggests the potential importance of identifying preceding infections in sepsis patients. These findings may support clinicians maintaining high awareness of prior infection and related microbiological findings when encountering patients with sepsis symptoms, as this knowledge may aid diagnosis and might be used to guide empirical treatment before blood culture findings are available. For example, sepsis patients with recently preceding pyelonephritis can probably benefit from antibiotic treatment covering Gram-negative bacteria to a greater extent. Additionally, the associations found in the present study might guide the follow-up prioritization of sepsis survivors discharged from hospitals to primary healthcare. For researchers, these findings may be useful to consider in studies examining sepsis treatment, care, and follow-up. For example, prospective clinical studies could examine whether knowledge of preceding infections in patients with sepsis can inform empirical treatment strategies. In addition, future studies could also examine whether the associations identified in this study can guide strategies to reduce the mortality following hospitalization for sepsis.

In conclusion, several sociodemographic factors were associated with sepsis mortality, and old age was a particularly strong risk factor. A preceding pyelonephritis was associated with lower sepsis mortality. These findings can be used to increase the awareness of potential risk factors for sepsis mortality and provide a foundation for clinical studies aiming at improving sepsis survival.

## Abbreviations

CCI, Charlson Comorbidity Index; CI, Confidence Interval; ICD, International Classification of Diseases; OR, Odds Ratio.

## Data availability

This study made use of several national registers and, owing to legal concerns, data cannot be made openly available. Further information (including accessibility to these data) regarding the nationwide registries is available from the Swedish National Board of Health and Welfare (https://www.socialstyrelsen.se/en/statistics-and-data/registers/) (registerservice@socialstyrelsen.se) and *Statistics Sweden (*https://www.scb.se/en/) (scb@scb.se). These authorities may, under certain circumstances, provide anonymized data to researchers without ethical approval. If this is not possible, ethical permission is needed (https://etikprovningsmyndigheten.se/) (registrator@etikprovning.se). The code used in the analysis can be provided upon reasonable request from Professor Kristina Sundquist.

## Authors' statement

All authors have approved the final version of the manuscript. The authors attest that all listed authors meet the authorship criteria and that no others meeting the criteria have been omitted.

## Credit author statement

Filip Jansåker: Conceptualization; methodology; validation; investigation; writing-original draft; writing-review & editing; visualization; project administration; funding acquisition.

Xinjun Li: Conceptualization; methodology; software; validation; formal analysis; investigation; data curation; writing-review & editing; visualization.

Per-Ola Forsberg: Conceptualization; methodology; validation; investigation; writing-review & editing; visualization.

Kristina Sundquist: Conceptualization; methodology; software; validation; investigation; data curation; resources; writing-review & editing; visualization; supervision; project administration.

Henning Stenberg: Conceptualization; methodology; validation; investigation; writing-original draft; writing-review & editing; visualization; supervision.

## Declaration of competing interest

The authors declare no conflicts of interest.

## Appendix Supplementary materials

Image, application 1
