# Supplementary material for: Risk factors for sepsis mortality: A national Swedish cohort study (1998–2018)
Source: Clinics (Sao Paulo). 2026 Jun 10;81:101022. doi: 10.1016/j.clinsp.2026.101022 (PMC13273217; doi:10.1016/j.clinsp.2026.101022)
Supplement: Supplementary file 1 [file mmc1.docx]

**CLINICS-D-25-01441**

**Supplementary material**

**Figure S1. Flow diagram (study population)**

**Table S1. ICD-10 codes and number of sepsis cases, 1998–2018**

**Table S2. Charlson Comorbidity Index of individuals with sepsis and 30-day mortality**

**Table S3. Number of individuals diagnosed with sepsis or septic shock (first event), time period (calendar years) of sepsis diagnosis, and 30-day all-cause mortality**

**Table S4. Association of individual sociodemographic factors and comorbidities with 30-day all-cause sepsis mortality, by country of origin**

**Table S5. Association of individual sociodemographic factors and comorbidities with 30-day all-cause septic shock mortality (n=2627), 1998–2018**

**Table S6. Association of sociodemographic factors and comorbidities with 90-day all-cause sepsis mortality**

**Statistic formulas**

**Figure S1** Flow diagram (study population).

**Table S1** ICD-10 codes and number of sepsis cases, 1998–2018.

| **Type of Sepsis** | **ICD-10 code** | **No.** | **%** |
| --- | --- | --- | --- |
| Other sepsis | A41 | 127,017 | 80.8 |
| Streptococcal sepsis | A40 | 20,500 | 13.0 |
| Severe sepsis | R65.1 | 3961 | 2.5 |
| Septic shock^a^ | R57.2 | 2627 | 1.7 |
| Salmonella sepsis | A02.1 | 958 | 0.6 |
| Sepsis (due to) (in) candidal | B37.7 | 854 | 0.6 |
| Sepsis (due to) (in) listerial | A32.7 | 342 | 0.2 |
| Sepsis (due to) Pasteurellosis | A28.0 | 332 | 0.2 |
| Sepsis (due to) (in) meningococcal | A39.2-4 | 211 | 0.1 |
| Sepsis (due to) Shigella | A03.9 | 175 | 0.1 |
| Sepsis (due to) brucellosis | A23.9 | 33 | 0.0 |
| Sepsis (due to) (in) extraintestinal yersiniosis | A28.2 | 31 | 0.0 |
| Sepsis (due to) (in) Erysipelothrix | A26.7 | 29 | 0.0 |
| Sepsis (due to) (in) actinomycotic | A42.7 | 29 | 0.0 |
| Sepsis (due to) (in) herpesviral | B00.7 | 23 | 0.0 |
| Sepsis (due to) (in) tularemia | A21.7 | 14 | 0.0 |
| Sepsis (due to) (in) melioidosis | A24.1 | 5 | 0.0 |
| Sepsis (due to) (in) anthrax | A22.7 | 2 | 0.0 |
| Sepsis (due to) (in) plague | A20.7 | 0 | 0.0 |
| Sepsis (due to) (in) gonococcal | A54.86 | 0 | 0.0 |
| **All above (**including septic shock) |  | **157,143** | **100.0** |

ICD-10, 10th revision of the International Classification of Diseases; No, Number of cases. The following ICD-10 diagnoses were not included in the study: O08.0 Sepsis following abortion (subsequent episode); O08.2 Sepsis following ectopic or molar pregnancy; O75.3 Sepsis during labor; O85 Puerperal sepsis; P36 (all codes) Bacterial sepsis of new-born; T80.2 Sepsis following infusion, therapeutic injection or transfusion; T81.4 Sepsis (due to) Infection following a procedure, not elsewhere classified; T88.0 Sepsis following immunization; and J95.02 Sepsis (due to) tracheostomy stoma.

^a^ Septic shock cases were excluded from main analyses and analyzed separately.

**Table S2** Charlson Comorbidity Index of individuals with sepsis and 30-days mortality.

|  | **The 9^th^ revision of the International Classification of Diseases (ICD-9) (before 1997)** | **The 10^th^ revision of the International Classification of Diseases (ICD-10)** | **Study population** | | **30-day mortality** |
| --- | --- | --- | --- | --- | --- |
| **Comorbidities** |  |  | **No** | **%** | **No** |
| Myocardial infarction | 410.x, 412.x | I21, I22, I25 | 9839 | 6.3 | 2211 |
| Congestive heart failure | 398.91, 402.01, 402.11, 402.91, 404.01, 404.03, 404.11, 404.13, 404.91, 404.93, 425.4 - 425.9, 428.x | I43, I50, I09.9, I11.0, I13.0, I13.2, I25.5, I42.0, I42.5, I42.6. I42.7, I42.8, I42.9, P29.0 | 12,679 | 8.1 | 3653 |
| Peripheral vascular disease | 093.0, 437.3, 440.x, 441.x, 443.1 - 443.9, 447.1, 557.1, 557.9, V43.4 | I70, I71, I73.1, I73.8, I73.9, I77.1, I79.0, I79.2, K55.1, K55.8, K55.9, Z95.8, Z95.9 | 6145 | 3.9 | 1544 |
| Cerebrovascular disease | 362.34, 430.x - 438.x | I60-I69, G45, G46, H34.0 | 11,986 | 7.6 | 2703 |
| Dementia | 290.x, 294.1, 331.2 | F00, F01, F02, F03, G30, F05.1, G31.1 | 4269 | 2.7 | 1448 |
| Chronic pulmonary disease | 416.8, 416.9, 490.x - 505.x, 506.4, 508.1, 508.8 | J40-J47, J60-J67, I27.8, I27.9, J68.4, J70.1, J70.3 | 8131 | 5.2 | 1808 |
| Rheumatic disease | 446.5, 710.0 - 710.4, 714.0 - 714.2, 714.8, 725.x | M05, M06, M32, M33, M34, M31.5, M35.1, M35.3, M36.0 | 4178 | 2.7 | 855 |
| Peptic ulcer disease | 531.x - 534.x | K25, K26, K27, K28 | 3427 | 2.2 | 802 |
| Mild liver disease | 070.22, 070.23, 070.32, 070.33, 070.44, 070.54, 070.6, 070.9, 570.x, 571.x, 573.3, 573.4, 573.8, 573.9, V42.7 | B18, K73, K74, K70.0, K70.3, K70.9, K71.3, K71.5, K71.7, K76.0, K76.2, K76.4, K76.8, K76.9, Z94.4 | 1556 | 1.0 | 264 |
| Diabetes without chronic complication | 250.0 - 250.3, 250.8, 250.9 | E10.0, E10.1, E10.6, E10.8, E10.9, E11.0, E11.1, E11.6, E11.8, E11.9, E12.0, E12.1, E12.6, E12.8, E12.9, E13.0, E13.1, E13.6, E13.8, E13.9, E14.0, E14.1, E14.6, E14.8, E14.9 | 9925 | 6.3 | 2004 |
| Diabetes with chronic complication | 250.4 - 250.7 | E10.2, E10.3, E10.4, E10.5, E10.7, E11.2, E11.3, E11.4, E11.5, E11.7, E12.2, E12.3, E12.4, E12.5, E12.7, E13.2, E13.3, E13.4, E13.5, E13,7, E14.2, E14.3, E14.4, E14.5, E14.7 | 4650 | 3.0 | 871 |
| Hemiplegia or paraplegia | 334.1, 342.x, 343.x, 344.0 - 344.6, 344.9 | G81, G82, G04.1, G11.4, G80.1, G80.2, G83.0, G83.1, G83.2, G83.4, G83.9 | 663 | 0.4 | 123 |
| Renal disease | 403.01, 403.11, 403.91, 404.02, 404.03, 404.12, 404.13, 404.92, 404.93, 582.x, 583.0 - 583.7, 585.x, 586.x, 588.0, V42.0, V45.1, V56.x | N18, N19, N12.0, N13.1, N03.2, N03.3, N03.4, N03.5, N03.6, N03.7, N05.2-N05.7, N25.0, Z49.0, Z49.1, Z49.2, Z94.0, Z99.2 | 5270 | 3.4 | 1183 |
| Any malignancy, including lymphoma and leukemia, except malignant neoplasm of skin | 140.x - 172.x, 174.x - 195.8, 200.x - 208.x, 238.6 | C00-C09, C10-C19, C60-C69, C20-C26, C30, C31, C32, C33, C34, C37, C38, C39, C40, C41, C43, C45, C46, C47, C48, C49, C50-C58, C70-C76, C81-C85, C88, C90-C97 | 19,781 | 12.6 | 4125 |
| Moderate or severe liver disease | 456.0 - 456.2, 572.2- 572.8 | I85.0, I85.9, I86.4, I98.2, K70.4, K71.1, K72.1, K72.9, K76.5, K76.6, K76.7 | 604 | 0.4 | 142 |
| Metastatic solid tumor | 196.x - 199.x | C77-C80 | 2823 | 1.8 | 661 |
| AIDS/HIV | 042.x - 044.x | B20, B21, B22, B24 | 71 | 0.0 | 7 |

AIDS/HIV, Acquired Immunodeficiency Syndrome/Human Immunodeficiency Virus.

**Table S3** Number of individuals diagnosed with sepsis or septic shock (first event), time period (calendar years) of sepsis diagnosis, and 30-day all-cause mortality.

|  | **Individuals** | **30-day all-cause mortality** | |
| --- | --- | --- | --- |
|  | **No.** | **No.** | **%** |
| **Sepsis** | 154,516 | 27,722 | 17.9 |
| Period: 1998–2005 | 62,365 (40.4)^*^ | 9719 (35.1) | 15.6 |
| Period: 2006–2012 | 69,836 (45.2)^*^ | 12,687 (45.8) | 18.2 |
| Period: 2013–2018 | 22,315 (14.4)^*^ | 5316 (19.2) | 23.8 |
| **Septic shock** | 2627 | 1413 | 53.8 |
| All | 157,143 | 29,135 | 18.5 |

**Table S4** Association of individual sociodemographic factors and comorbidities with 30-day all-cause sepsis mortality, by country of origin.

|  | **Born in Sweden** | | | | **Foreign-born** | | | |
| --- | --- | --- | --- | --- | --- | --- | --- | --- |
| **Covariates** | **OR** | **95% CI** | | **p-value** | **OR** | **95% CI** | | **p-value** |
| **Age** (ref. age 18–44 years) |  |  |  |  |  |  |  |  |
| 45–64 | 2.96 | 2.59 | 3.39 | <0.0001 | 3.60 | 2.36 | 5.49 | <0.0001 |
| 65–84 | 6.47 | 5.68 | 7.36 | <0.0001 | 7.98 | 5.30 | 12.03 | <0.0001 |
| ≥ 85 | 12.64 | 11.09 | 14.39 | <0.0001 | 17.45 | 11.51 | 26.46 | <0.0001 |
| **Male sex** (ref. female) | 1.01 | 0.98 | 1.04 | 0.565 | 1.09 | 1.00 | 1.20 | 0.0636 |
| **Educational level** (ref. >12 years) | 1.08 | 1.04 | 1.13 | <0.0001 | 1.12 | 1.01 | 1.25 | 0.0325 |
| **Family income** (ref. high) | 1.19 | 1.15 | 1.23 | <0.0001 | 1.06 | 0.94 | 1.19 | 0.3612 |
| **Region of residence** (ref. large cities) | 1.08 | 1.05 | 1.12 | <0.0001 | 1.15 | 1.04 | 1.26 | 0.0046 |
| **Marital status** (ref. married/cohabiting) | 1.18 | 1.14 | 1.21 | <0.0001 | 1.11 | 1.01 | 1.22 | 0.0247 |
| **Charlson Comorbidity Index** (ref. low, 0 p) |  |  |  |  |  |  |  |  |
| Moderate (1–2 p) | 1.33 | 1.29 | 1.37 | <0.0001 | 1.35 | 1.23 | 1.48 | <0.0001 |
| High (≥3 p) | 1.92 | 1.62 | 2.28 | <0.0001 | 2.06 | 1.28 | 3.32 | 0.0029 |
| **Severe mental disorders** (ref. no diagnosis) | 0.95 | 0.84 | 1.07 | 0.3693 | 0.89 | 0.62 | 1.26 | 0.4987 |

OR, Odds Ratio; CI, Confidence Interval. Full model, adjusted for all covariates. Analysis excluding those diagnosed with septic shock (n=2627).

**Table S5** Association of individual sociodemographic factors and comorbidities with 30-day all-cause septic shock mortality (n=2627), 1998–2018.

| **Covariates** | **OR** | **95% CI** | | **p-value** |
| --- | --- | --- | --- | --- |
| **Age** (ref. age 18–44 years) |  |  |  |  |
| 45–64 | 5.78 | 2.03 | 16.45 | <0.0001 |
| 65–84 | 13.95 | 4.95 | 39.31 | <0.0001 |
| ≥85 | 36.07 | 12.65 | 102.83 | <0.0001 |
| **Male sex** (ref. female) | 0.88 | 0.75 | 1.04 | 0.1231 |
| **Educational level** (ref. >12 years) | 1.21 | 0.98 | 1.49 | 0.0745 |
| **Family income** (ref. high) | 1.14 | 0.95 | 1.37 | 0.1694 |
| **Region of residence** (ref. large cities) | 0.99 | 0.84 | 1.17 | 0.9281 |
| **Marital status** (ref. married/cohabiting) | 1.28 | 1.08 | 1.51 | 0.0051 |
| **Country of origin** (ref. Sweden) | 1.16 | 0.89 | 1.52 | 0.2767 |
| **Charlson Comorbidity Index** (ref. low, 0p) |  |  |  |  |
| Moderate (1–2 p) | 1.35 | 1.15 | 1.59 | 0.0004 |
| High (≥3 p) | 1.01 | 0.45 | 2.29 | 0.9808 |
| **Severe mental disorders** (ref. no diagnosis) | 0.64 | 0.35 | 1.15 | 0.1344 |

OR: Odds ratio; CI: Confidence interval. Full model, adjusted for all covariates.

**Table S6** Association of sociodemographic factors and comorbidities with 90-day all-cause sepsis mortality.

| **Covariates** | **OR** | **95% CI** | | **p-value** |
| --- | --- | --- | --- | --- |
| **Age** (ref. age 18–44 years) |  |  |  |  |
| 45-64 | 2.97 | 2.68 | 3.29 | <0.0001 |
| 65–84 | 6.28 | 5.69 | 6.93 | <0.0001 |
| ≥85 | 12.14 | 10.99 | 13.42 | <0.0001 |
| **Male sex** (ref. female) | 1.03 | 1.01 | 1.06 | 0.0145 |
| **Educational level** (ref. >12 years) | 1.06 | 1.03 | 1.10 | 0.0002 |
| **Family income** (ref. high) | 1.12 | 1.09 | 1.16 | <0.0001 |
| **Region of residence** (ref. large cities) | 1.08 | 1.06 | 1.11 | <0.0001 |
| **Marital status** (ref. married/cohabiting) | 1.15 | 1.12 | 1.18 | <0.0001 |
| **Country of origin** (ref. Sweden) | 0.93 | 0.89 | 0.97 | 0.0005 |
| **Charlson Comorbidity Index** (ref. low, 0 p) |  |  |  |  |
| Moderate (1–2 p) | 1.44 | 1.40 | 1.47 | <0.0001 |
| High (≥3 p) | 2.76 | 2.40 | 3.16 | <0.0001 |
| **Severe mental disorders** (ref. no diagnosis) | 0.85 | 0.77 | 0.94 | 0.0015 |

OR, Odds ratio; CI, Confidence interval. Full model, adjusted for all covariates. Analysis excluding those diagnosed with septic shock (n=2627).

**Statistic formulas**

Adjusted *Odds Ratio* (OR) for sepsis mortality associated with a predictor variable in a multivariate logistic regression model was calculated as: OR=e^β^. Where ‘e’ is the base of the natural logarithm (approximately 2.71828) and ‘β’ (beta) is the coefficient (log odds) for the independent variable in the logistic regression equation, after controlling for other variables (confounders) in the model. *Mortality rate* (%) = Number of morality cases/study population*100, with 95% Confidence Interval (95% CI) = mortality rate ± 1.96*mortality rate/$\sqrt{number of mortality cases}$.
